# Supplementary material for: Clinical outcomes of corneal neurotization using sural nerve graft in neurotrophic keratopathy
Source: PLoS One. 2023 Nov 28;18(11):e0294756. doi: 10.1371/journal.pone.0294756 (PMC10684005; doi:10.1371/journal.pone.0294756)
Supplement: S1 Table — Data of the enrolled patients in the study. (DOCX) [file pone.0294756.s001.docx]

**S1 Table. Study’s underlying data**

Patient data of recruited eyes at the baseline and at serial follow up visits.

**The authors also confirm that the study has been registered under Clinical Trial Registry, India with Reference no- CTRI/2021/10/037280.**

| S. No. | Age/  Sex | Etiology | Duration of disease (Years) | Mackie stage | Follow up | BCVA  (preop) | Central corneal sensation (Preop score) | SBNFD  (pre-op values) | SBNFL  (Pre-op values) | BCVA  (Last visit) | Central corneal sensation (last visit) | SBNFD  (Last visit) | SBNFL  (Last visit) | Mackie  stage  (Last visit) |
| --- | --- | --- | --- | --- | --- | --- | --- | --- | --- | --- | --- | --- | --- | --- |
| 1 | 69/F | HSV | 3 | 2 | 1yr | 1 | 0 | 0 | 0.67 | 0.6 | 4 | 6.57 | 15.31 | 1 |
| 2 | 70/M | HZV | 4 | 2 | 1yr | 1.8 | 0 | 0 | 2.79 | 1 | 0.5 | 2.04 | 7.61 | 1 |
| 3 | 71/M | HSV | 3 | 2 | 1yr | 0.8 | 0 | 0 | 5.17 | 0.2 | 2 | 2.69 | 13.14 | 1 |
| 4 | 25/F | FNP | 2 | 3 | 1yr | 1.8 | 0 | 0 | 7.1 | 1.5 | 0.5 | 1.63 | 11.09 | 2 |
| 5 | 40/F | HSV | 3 | 2 | 1yr | 0.8 | 0.5 | 0 | 1.88 | 0 | 4.5 | 8.24 | 18.37 | 0 |
| 6 | 26/M | FNP | 2 | 2 | 1yr | 0.5 | 0 | 0 | 1.3 | 0 | 2.5 | 8.47 | 14.74 | 1 |
| 7 | 30/M | HSV | 3 | 3 | 9m | 1.9 | 0 | 0 | 4.53 | 1.9 | 0.5 | 0 | 6.46 | 2 |
| 8 | 28/M | FNP | 2 | 3 | 9m | 1.9 | 0 | 0 | 2.63 | 1.9 | 0.5 | 0 | 5.13 | 2 |
| 9 | 26/M | FNP | 2 | 3 | 9m | 1.3 | 0 | 0 | 3.2 | 1 | 0 | 0 | 6.98 | 2 |
| 10 | 74/M | HSV | 3 | 3 | 6m | 1.9 | 0 | 0 | 2.3 | 1.9 | 0 | 0 | 2.59 | 3 |
| 11 | 31/F | HSV | 2 | 2 | 6m | 1.2 | 0.5 | 0 | 2.64 | 1 | 0 | 0.54 | 5.16 | 1 |
