# Supplementary material for: Clinical outcomes of corneal neurotization using sural nerve graft in neurotrophic keratopathy
Source: PLoS One. 2023 Nov 28;18(11):e0294756. doi: 10.1371/journal.pone.0294756 (PMC10684005; doi:10.1371/journal.pone.0294756)
Supplement: S1 File — Proposed protocol for the ethical approval of our study titled Clinical outcomes of corneal neurotization using sural nerve graft in neurotrophic keratopathy. (DOC) [file pone.0294756.s003.doc]

***Clinical Outcomes of Corneal Neurotization using Sural Nerve Graft in Neurotrophic Keratopathy***

**THE POSTGRADUATE INSTITUTE OF MEDICAL
EDUCATION AND RESEARCH
CHANDIGARH**

**Junior Resident**

**DR. AMAN KALIA CHIEF GUIDE**

**DR. MANU SAINI**

ASST. PROFESSOR

DEPARTMENT OF OPHTHALMOLOGY

PGIMER CHANDIGARH

**CO GUIDE CO GUIDE**

**DR. ARUN K. JAIN DR. AMIT GUPTA**

PROFESSOR PROFESSOR

DEPT. OF OPHTHALMOLOGY DEPT. OF OPHTHALMOLOGY

PGIMER CHANDIGARH PGIMER CHANDIGARH

**CO GUIDE CO GUIDE**

**DR. SUNIL GABA DR. CHINTAN MALHOTRA**

ADDITIONAL PROFESSOR ADDITIONAL PROFESSOR

DEPT.OF PLASTIC SURGERY DEPT. OF OPHTHALMOLOGY

PGIMER CHANDIGARH PGIMER CHANDIGARH

**CO GUIDE**

**DR. MANPREET SINGH**

ASSISTANT PROFESSOR

DEPT. OF OPHTHALMOLOGY

PGIMER CHANDIGARH

**TABLE OF CONTENTS** **PAGES**

01.Abbreviations 4

02.Introduction 5-9

03.Review of Literature 10-14

04.Aim of study 15

05.Materials and Methods 16-25

06.Ethical Justification 26-27

07.Statistical analysis 28

08.Patient information sheet( English) 29-33

09.Patient information sheet(Hindi) 34-37

10.Patient information sheet(Punjabi) 38-41

11.Patient Consent Form (English) 42-43

12.Patient Consent Form (Hindi) 44-45

13.Patient Consent Form (Punjabi) 46

14.Investigator certificate 47

15.Patient Performa 48-50

16.Annexures 51

17.Bibliography 52-55

**ABBREVIATIONS**

BCVA Best-corrected visual acuity

CBA Cochett Bonnet Anesthsiometer

CCK Cholecystokinin

CGRP Calcitonin Growth-Related Peptide

DNA Deoxyribonucleic Acid

FNP Facial Nerve Palsy

FTBUT Fluorescein Tear film Break Up Time

IVCM In vivo Confocal Microscopy

KP Keratoplasty

MICN Minimally Invasive Corneal neurotisation

NK Neurotropic Keratopathy

NSAIDs Non-steroidal Anti-inflammatory Drugs

PED Persistent epithelial defect

SP Substance P

**INTRODUCTION**

Corneal innervation plays a vital role in epithelial integrity, proliferation, wound healing, DNA synthesis, and collagen expression. The sub-basal nerve plexus along with stromal keratocytes secrete several neuropeptides, which facilitate cell mitogenesis, migration, DNA synthesis, neurite extension, and survival of keratocyte proliferation.^1^ These diffusible neuropeptides are believed to regulate epithelial stem cells, stimulate the epithelial growth, proliferation, differentiation, and the production of collagen type VII.^2,3^

Neuropeptides contained in sensory nerves are involved in the trophic effects; substance P (SP), calcitonin gene-related peptide (CGRP), and cholecystokinin (CCK) have been identified immunocytochemically in corneal nerve fibers. There is evidence that neuropeptide depletion by capsaicin in new-born mouse induces neuroparalytic like corneal changes.^3^

Corneal sensory innervation requires maintaining ocular surface homeostasis, including blink reflex, corneal wound healing, tear production, and limbal stem cell function,^4,5^ and its impairment lead to a degenerative corneal condition known as Neurotrophic keratopathy (NK). NK results in the breakdown of the corneal epithelium, affecting the health and integrity of the tear film, epithelium, and stromal cells of the cornea.^6^ In absence of protective sensation, repetitive minor corneal injury can ultimately lead to permanent blindness from chronic corneal scarring.^7^

Clinical presentation of NK ranges from subtle corneal surface irregularities to corneal melting and perforation. NK is usually graded in three different stages in accordance with the “Mackie classification”^8^

**MACKIE cLASSIFICATIOn**

| **STAGE** | - **CLINICAL FEATURES** | **TREATMENT** |
| --- | --- | --- |
| **1** | - Inferior palpebral conjunctiva staining with Rose Bengal (earliest sign) - Decreased TBUT - Increased mucous viscosity - Punctate epithelial fluorescein staining - Scattered small facets of dried epithelium(Gaule spots) | Lubricating Eye Drops |
| **2** | - Epithelial defect surrounded by a rim of loose epithelium - Stromal swelling with Descemet membrane folds - Edges of defect become smooth & rolled with time - Rare anterior chamber inflammatory action. - Recurrent PED | Artificial tears, lubricant ointments  Therapeutic soft contact lenses or patching  Topical autologous serum application,  Amniotic membrane grafting,  Tarsorrhaphy or botulinum induced ptosis  Topical Nerve Growth Factor application |
| **3** | - Corneal ulcer - Stromal Melting /Lysis - Corneal Perforation | Therapy for Stage 1 & 2 with  N-acetylcysteine,oral tetracycline,and medroxyprogesterone |

Stage I is characterized by hyperplasia and/or irregularity of the epithelium, evolving to punctate keratopathy, corneal edema, neovascularization, stromal scarring.

Stage II is defined by a recurrent or persistent epithelial defect (PED), most commonly in the superior half of the cornea. The PED is usually oval in shape and its margins are characteristically smooth and rolled due to impaired epithelial healing.

In stage III, stromal involvement leads to corneal ulcer, melting, and perforation.^8^

There have been histological alterations seen in the cornea of patients with NK, including thinning/disruption of the epithelial layer, cytoplasmic swelling of epithelial cells, loss of microvilli, disorganization of Bowman’s membrane, stromal melting/scarring and corneal neovascularization. The conjunctiva is also involved with a reduction in goblet cell density and cell-surface microplicae. Evidence in animal models suggests that NK may also affect corneal neovascularisation^9^ and stem cell populations ^10^.

Various aetiologies have been implicated in the manifestation of NK. In our study, we are including herpetic keratitis, chemical eye injury, injury/damage to the trigeminal nerve following intracranial space-occupying lesion such as acoustic neuroma and/or neurosurgical procedures that damage the branch of the trigeminal ophthalmic nerve.

Various medical and surgical therapeutic strategies are described for unilateral or bilateral NK. However, most treatments to date aim at protecting the corneal surface and promoting corneal epithelial healing, instead of addressing underlying corneal anesthesia. ^11,12^

In 2009, Terzis et al^13^ described an innovative surgical technique to reinnervate the ipsilateral aesthetic cornea using the contralateral supraorbital and supratrochlear nerves named corneal neurotization. They proposed that the mechanism of corneal neurotization was related to direct sprouting from the healthy contralateral transplanted nerves into the anesthetic cornea.

Fung et al^14^ (from the same group as Elbaz et al) observed regeneration of corneal nerves after corneal neurotization using in vivo confocal microscopy (IVCM). Terzis et al ^13^and Elbaz et al^15^ postulated that the improvement in corneal sensation was attributed to axonal regeneration of the nerve graft fascicles inserted around the corneal limbus with subsequent growth into the corneal stromal or subepithelial level.

Reinnervation follows a predictable timeline, with neurotization of the cornea near the limbus visible at 8 weeks postoperatively, neurotization of the superficial central corneal seen at 3 to 7 months postoperatively, and complete neurotization including the basal layers of the central cornea is seen between 6 months and 2 years postoperatively. Although this timeline is affected by factors such as the patient’s age and health before surgery.^16-18^

Variety of nerve autografts have been mentioned in the literature including the sural nerve, lateral antebrachial cutaneous nerve, and great auricular nerve. Selection is based on availability at the surgical site, the number of fascicles, and graft length requirement. If a short inter-positional segment is required, the great auricular nerve is often available locally and may deliver graft lengths of up to 7 cm. Sural nerve graft tends to be preferred choice as it has the highest percentage of nerve fibers (fascicles), being superficially located, easy to harvest and the desired length can be obtained with minimum donor site morbidity^19^

Hence, through this study, we sought to determine the effectiveness of Corneal Neurotization using Sural Nerve Graft co-apted to the contralateral supratrochlear nerve in unilateral Neurotrophic Keratopathy eyes in Indian population.

**REVIEW OF LITERATURE**

Surgical corneal neurotisation may be done either by direct nerve transfer or by using an interpositional nerve graft coapted to a healthy donor nerve. The concept of corneal neurotisation was introduced by Samii in 1972 and described in his textbook in 1981.^20,21^ He described anastomosing the major occipital nerve to the proximal ophthalmic nerve using a sural nerve graft placed in the subgaleal plane. This was achieved by dissection of the occipital nerve, followed by a frontal craniotomy approach to expose and enter the orbital roof. The ophthalmic nerve was exposed, transected and its distal stump anastomosed with the sural nerve graft. Three patients have been described in whom the trophic function of the cornea improved, but not a full recovery of sensation was noted.

**DIRECT NEUROTIZATION**

First successful Direct nerve transfer was done by Dr. Terzis and colleagues in 2009.^13^ They conducted the procedure of direct corneal neurotisation in cases of unilateral facial nerve palsy (FNP) with corneal anesthesia, as part of a staged approach to FNP reanimation in patients with combined FNP and ipsilateral trigeminal nerve involvement. Their technique involved a nerve transfer of the contralateral supratrochlear and supraorbital nerves to directly neurotize the neurotrophic cornea. The contralateral intact supraorbital and supratrochlear nerves were carefully dissected and were used to restore sensation to the affected cornea. All six patients in the study reported subjective sensibility between 6 months and 1 year, the average time to objective sensibility was 2.80+/-2.17 years.^13^

Allevi et al^22^ reported a case in 2014 of a patient with vision +1.0 (6/60) who had previously undergone facial motor reanimation followed by direct corneal neurotisation using the technique described by Terzis et al. The procedure involved transfer of the contralateral supraorbital and supratrochlear nerves to four perilimbal cardinal points around the affected cornea by the above technique. Corneal sensation improved, spontaneous blinking recovered, and the peripheral cornea became clear, especially in the superior, temporal, and nasal quadrants. Six months after corneal neurotisation, the patient received penetrating Keratoplasty (KP). Follow-up of KP was uneventful, with a clear cornea and no epithelial defects; the patient progressively gained vision up to 0.7 (6/30) with correction by 6 months post-KP.

**Ipsilateral nerve transfer technique**

In 2016, Jacinto et al reported the use of the ipsilateral supraorbital nerve for direct neurotisation to treat neurotrophic keratopathy due to direct damage to the long ciliary nerves. Using a hemicoronal incision and flap, three branches of the ipsilateral supraorbital nerve, 6 cm in length (from the supraorbital notch) were transferred, through to the sub-Tenon’s space to place them 360 degrees perilimbal to reach 6 o’clock. The patient reported Continued ocular comfort and improved vision.^23^

Ipsilateral nerve transfer is a good option for direct neurotisation where neurotrophic keratopathy is due to long ciliary nerve damage and ipsilateral supratrochlear and supraorbital nerves are intact. But, this approach traditionally required a large bi-coronal, or hemicoronal incision and flap. Dissecting out the supratrochlear and supraorbital nerves with a significant length (approximately 12 cm to allow transfer to the contralateral cornea and its lateral most limbus, or at least 6 cm for the ipsilateral cornea) involves time-consuming careful dissection under high magnification. Reflection of the coronal flap prevents from concurrently preparing the ocular surface for the insertion of nerve fascicles at the same time as harvesting of the nerves, thus prolonging the procedure. It is also not applicable for rarer, bilateral cases of FNP where a bilateral ophthalmic division of trigeminal nerve involvement exists.

In June 2016, the use of the ipsilateral infraorbital nerve to directly neurotize an anesthetic cornea in a patient with ipsilateral trigeminal ophthalmic division damage only was presented at the Congress of the Italian Society of Stem Cells and Ocular Surface.^24^ At the same time as masseteric-to-facial nerve anastomosis, the infraorbital nerve was dissected from its foramen to lips and transected distally. The nerve was reflected superiorly and tunneled to the conjunctival fornix.

In 2018, Leyngold et al ^25^reported the use of the contralateral supraorbital nerve, harvested through a combined transpalpebral and endoscopic forehead approach, for direct neurotisation to treat neurotrophic keratopathy following herpes zoster infection.

Ting et al ^26^ in 2018 reported two cases of direct corneal neurotisation for severe unilateral neurotrophic keratopathy secondary to cerebellopontine angle meningioma. The original surgical technique described by Terzis et al in 2009 was used. Two patients have been studied, one patient had significant improvement in corneal sensation and identifiable subbasal and stromal corneal nerves in 2^nd^ patient IVCM failed to identify subbasal and stromal corneal nerves and improvement in corneal sensation

**INTERPOSITONAL NERVE GRAFT TECHNIQUE**

The technique and principles of this strategy were developed in Toronto and reported by Elbaz et al^15^and Bains et al ^27^ to use a reversed sural nerve graft, coapted to the supratrochlear or supraorbital nerve and nerve fascicles sutured subconjunctivally to the perilimbal region. A transverse sub-brow incision was used to access the supratrochlear nerve and end-to-side coaptation of the sural nerve graft was achieved by creating an epineural window. Fibrin glue and 10-0 nylon sutures were used for coaptation. In unilateral cases, the contralateral supratrochlear nerve was used, requiring subcutaneous tunneling of the reversed nerve graft over the nasal bridge to the perilimbal area of the cornea. The epineurium was removed distally and the individual fascicles were separated. Approximately five fascicles were then placed around the entire limbal circumference and secured to the sclera with 10-0 nylon sutures. In both studies eyes with previous corneal anesthesia, all eyes regained corneal sensation after corneal neurotization. Elbaz et al reported markedly improved corneal sensation 6 months postsurgery in 2 patients & within 7.5 months in the third patient. Bains et al reported the establishment of protective corneal sensation in all patients by 6 months

Sepehripour et al ^28^ reported this procedure in a 2-year-old using a reversed sural nerve graft coapted end-to-end to a single fascicle (divided distally) of the contralateral supratrochlear nerve, exposed as it exited its notch. Six fascicles of the nerve graft were tunneled subconjunctivally and sutured to the perilimbal sclera

Weis et al ^29^ published their results in six adult patients using this technique. They coapted the sural nerve graft to the supraorbital nerve only when they exposed a small supratrochlear nerve. The nerve graft was coapted to the ipsilateral side if the skin sensation along the distribution of supraorbital and supratrochlear nerves was intact, otherwise, the graft was coapted to the contralateral side. The bony supraorbital canal was unroofed and the supraorbital nerve was coapted proximally in the orbit if the supraorbital nerve was found to have divided within the canal.  All patients had improved corneal sensation within half a year following the procedure. Five patients (83%) demonstrated improved visual acuity. Of these patients, three (50%) had improved visual acuity with no further surgical intervention. One patient underwent cataract extraction 13 months after neurotization and another underwent amniotic membrane grafting 17 months after neurotization.

A prospective cohort study was conducted at The Hospital for Sick Children, Toronto, Canada by Catapano et al^30^ between November 2012 & February 2017 in which 19 eyes of 16 patients underwent Minimally Invasive Corneal Neurotization (MICN). Functioning sensory nerves on the face were transacted and coapted with the sural nerve graft. The nerve graft after separating fascicles was tunneled into the subconjunctival space of the affected eye(s). In the first seven eyes, the fascicles were laid in the perilimbal subconjunctival space, in the rest of the eyes, the ends of nerve fascicles were inserted into peripheral corneal stroma via a partial thickness corneoscleral tunnel incision. Episodes of corneal epithelial defects after MICN was significantly reduced (21% vs 89%, respectively p<0.0001). Four eyes had to undergo keratoplasty after MICN which regained sensation and fully re-epithelialized.

Neurotization is a budding revolutionary technique that shows promise of a cure for neurotrophic corneas.

**AIM OF THE STUDY**

To evaluate the efficacy of corneal neurotization using sural nerve graft coaptation to the contralateral supratrochlear nerve in patients with unilateral neurotrophic keratopathy and corneal anesthesia.

**MATERIALS AND METHODS**:

Study Design: Prospective, interventional study.

Place of study:

Patients will be recruited from the cornea clinic of Advanced eye centre, Postgraduate Institute of Medical Education and Research, Chandigarh. They will be subjected to a complete ophthalmological examination, relevant systemic examination, and investigation before the surgical procedure.

Study Duration: Recruitement from February 2020 to December 2021

Sample size:

The number of patients coming to Advanced Eye Centre with NK on an average is 0-1 per month, this is based on the patient record in the department for the year 2019. Keeping in view the number of patients a total of 11 patients with Unilateral Neurotrophic Keratopathy failed to respond with medical measures will be recruited in the time available to conduct the intervention with follow up.

Sampling Technique:

All the patients coming to AEC with Unilateral Neurotrophic Keratopathy will be recruited in our study following certain Inclusion and Exclusion criteria as follows:

**Inclusion criteria:**

**∙**Consenting to participate in the study

**∙**Age more than 18 years

**∙**Patients with Corneal anesthesia caused by viral keratitis, damage to the trigeminal nerve following intracranial space-occupying lesions such as acoustic neuroma, neurosurgical procedures that damaged the ophthalmic branch of Trigeminal nerve.

Procedure will be recommended to the patients with visually significant NK showing no response to the standard medical treatment

**∙**Patients willing to follow up

**Exclusion criteria:**

**∙**Associated lid malposition

**∙**Incomplete follow up

**∙**Patient with diabetes, leprosy or peripheral neuropathy

**∙**History of previous corneal surgery

**Preoperative workup**:

A detailed history along with demographic details will be taken from all the recruited patients i.e. presenting visual complaints with their respective duration, any history suggestive of other co-existing ophthalmological diseases.

• Best-corrected visual acuity (BCVA) of the patients will be recorded using the Snellen visual acuity chart at baseline and all follow-up visits.

• Anterior segment examination using slit-lamp will be done in all patients at baseline and all follow-up visits along with fluorescein staining.

• Posterior segment findings will be noted with the slit-lamp biomicroscopy using a +90 D dioptre lens and B scan ultrasound will be performed in eyes with media haze obscuring posterior segment visualization.

Ocular surface evaluation tests

**·**Schirmer’s test 1

**·**Corneal sensations threshold measurement using Cochett Bonnet Anesthsiometer (CBA)

**·**Fluorescein tear film break up time (FTBUT)

**·**Ocular surface staining and

**·**Confocal Microscopy will be performed for the corneal sub-basal nerve fiber layer imaging

The description of the tests to be done is as follows:

**Schirmer’s test 1:**

Schirmer’s test-1will be performed by placing a sterile, commercially available, 35 x 5 mm paper strip at the junction of middle and lateral one third under the inferior eyelid margin to avoid irritation to the cornea. After 5 minutes with eyes closed, the extend of wetting will be measured by referring to the ruler provided by the manufacturer on the envelope containing the strips.

A value less than 5 mm after 5 minutes is very specific (90%) but only 2% sensitive for diagnosing dry eye.^3^

**Corneal Sensitivity:**

Central corneal sensitivity will be measured by the Cochet-bonnet Anesthesiometer (CBA) 0.12mm diameter nylon filament. The CBA stimulates the corneal nerves by direct contact. This is achieved by gently pressing a nylon thread against the anterior corneal surface. The longest thread length will be used first (6 cm) since this provides the lowest stimulus intensity. Only if the subject is unable to detect the thread stimulus at this length, it is shortened in length (by 0.5 cm steps) until the stimulus was felt. The criteria for threshold were the filament length which gives a 50% positive response from four stimulus presentations.^32^

The threshold to stimulation with the Cochet-Bonnet will be measured in length of nylon filament (cm). The manufacturer of the Cochet-Bonnet aesthesiometer provides a calibration table that enables the conversion of nylon filament length to pressure exerted on the eye.

**Fluorescein Tear Film Break Up Time :**

Fluorescein tear film break up time (FTBUT) is defined as the interval between the last blink and the first appearance of a random black spot in the Fluorescein-stained tear film.

Procedure **-** All measurements will be made in a quiet, dimly lit examination room of relatively constant temperature and humidity. For the TBUT test, the subject will be seated at the slit lamp with the chin on the chin-rest and the forehead firmly pressed against the forehead rest. A wide beam (full aperture) will be used so that the whole cornea is moderately illuminated, and viewed with 10× magnification. Tear film stability test will be performed by using fluorescein – impregnated strips with a drop of non-preserved saline solution, placed in the lower conjunctival sac. The patient will be asked to blink three-five times after fluorescein application to distribute the fluorescein, and then to look `normally' directly ahead and try not to blink for as long as possible. Subjects will be instructed to blink when any feeling of discomfort is felt, to avoid reflex tearing. A stopwatch will be started immediately after the last blink and stopped at the first appearance of random black spot(s) or streak(s) or hole in the precorneal film and this time interval (measurement) will be recorded. ^33^ Normal FBUT is 10 seconds or more. Values less than 5 seconds are indicative of significant dry eye disease. ^34^

**Ocular Surface Staining:**

**Conjunctival Staining:**

Conjunctival staining will be evaluated with 1% lissamine green staining of temporal and bulbar conjunctiva (each further subdivided into 3 regions). Lissamine green determine the relative staining characteristics of the nasal and temporal conjunctiva.

Procedure **–**A 1% solution or filter paper strip impregnated with lissamine green will be used to introduce the dye into the tear film. The patient will be asked to blink to distribute the dye. The ocular surface will be scanned using the white light of the slit lamp. The extent and intensity of the stain will be assessed.

**Corneal Staining:**

Corneal staining will be evaluated using Fluorescein staining graded in the central, nasal, superior, temporal, and inferior region. Fluorescein stains when there is cellular membrane disruption or cell-cell junction loss and therefore useful in assessing the intactness of the epithelium barrier. Pseudostaining may occur when the dye pools in the indented, but healthy epithelium. Because fluorescein diffuses rapidly into the corneal stroma when there is a loss of epithelial integrity, the ability to see punctuate staining is lost after a short time period (1 to 3 minutes). Therefore it will be essential to assess staining as soon as possible after fluorescein instillation.

Procedure **–** A single drop of sterile non-preserved saline will be applied onto the fluorescein – impregnated strip. The drop will be allowed to just saturate the tip of the paper strip and the excess will be shaken off. The lower lid will be pulled down and the tip of strip touched gently on the inferior palpebral conjunctiva. The patient will be asked to gently close and roll the eyes around to adequately distribute the dye across the ocular surface. Using a blue exciter filter over a white light source, each eye will be examined in turn observing the staining pattern.

**Confocal Microscopy:**

Confocal microscopy will be carried out in all recruited eyes using NIDEK Technologies Srl, Italy Confoscan 4, central corneal images were taken, in automatic gain mode using the standard setting of 4 passes, with a scanning range of 200 µm to image the anterior layers of the cornea i.e. epithelium, sub-basal nerve fiber layer, stromal keratocytes at 40X magnifications .^34^

Procedure –One drop of lubricating viscous gel will be placed on the microscope objective piece which had a similar refractive index to the cornea. The instrument will be moved to a backward position. After topical anesthesia with one drop, the patient’s head will be positioned on an adjustable headrest. The confocal microscope with the 40X objective placed 1– 1.5mm above the apex of the corneal center, the patient will be asked to look into the light, so that the optical center aligned with a lateral accuracy of probably less than 1mm. Then the microscope will be brought into optical contact with the cornea by manual advancement of the micrometer – controlled Z drive. From this point, all further x-y-z movements of the instrument will then be controlled from the real-time picture displayed on the monitor. Images will be recorded, and quickly checked to ensure that the required images had been captured and are of sufficient quality for investigation. If satisfactory images are not obtained, the procedure will be repeated to get the desired images.^35^

Each eye will be scanned three times through its entire depth. For each eye, two best images will be selected for analysis. Out of which best one containing the maximum number of nerves imaged at the sub-basal plexus level will be selected for analysis.

Sub-basal nerve fiber layer will be identified as unmyelinated fiber bundles consist of straight and beaded fibers that course in the basal aspect of the basal epithelial cell layer, sub-basal nerve bundles are easily seen using confocal microscopy. Sub-basal nerve fiber layer image analysis will be done in a masked manner using custom NIH image J software from the web site “http://www.imagescience.org/meijering/software/neuronj/, freely downloaded from the internet. The tracing of subbasal nerves will be performed using Neuron J, a semi-automatic Image J plugin to facilitate the tracing and quantification of elongated image structures. Then the total nerve number, total length/frame of subbasal nerves will be measured automatically.

A subbasal nerve fiber bundle will be counted as a separate nerve if the length of its visible portion, including its appearance in adjacent images in some cases, longer than 50 µm. Nerve branches longer than 50 µm will be counted as separated nerves. The total number of subbasal nerves and branches will be recorded as the mean number per scan for each cornea.^36^

The mean subbasal nerve layer density will be calculated as the total length of all main nerves and their branches divided by area of standard frame size.

**Surgical Methodology**

Sural nerve graft will be harvested by the plastic surgeon. Coaptation of one end of the sural nerve graft to the supratrochlear nerve and transplantation of another end to the corneal limbus will be done by the ophthalmologist (chief guide) Dr. Manu Saini.

The procedure will be performed under GA. It will take approximately 2 hours. The patient will be admitted 1-2 days before scheduled surgery to undergo necessary ocular investigations and pre-anesthesia checkups.

The sural nerve to be used for corneal neurotisation is best identified approximately 2 cm posterior to the lateral malleolus and approximately 2 to 3 cm proximal. In this area, the nerve has not undergone significant branching. A longitudinal incision will be made in the region above. If the small saphenous vein is visible, the nerve or a branch of it is close by. The nerve will be traced proximally with a longitudinal incision as required, by gently pulling the nerve at the distal incision, the locations of proximal incisions will be determined. After the necessary exposition, the nerve will be cut proximally and distally and placed on moist gauze. The donor site will be closed with subcutaneous sutures of 4-0 vicryl and the skin will be closed with 3-0 nylon or staples.

A transverse sub-brow incision will be used to access the supratrochlear nerve and end-to-side coaptation of the sural nerve graft will be achieved by creating an epineural window. A reversed sural nerve graft will be coapted to the contralateral supratrochlear nerve and nerve fascicles sutured subconjunctivally to the perilimbal region, through the subcutaneous tunnel over the nasal bridge. Fibrin glue and 10-0 nylon sutures will be used for coaptation. The epineurium will be removed distally and the individual fascicles will be separated. Approximately five fascicles will be then placed around the entire limbal circumference and secured to the sclera with 10-0 nylon suture

The patient will be discharged on the next postoperative day after follow up examinations and will be called on scheduled follow up visits


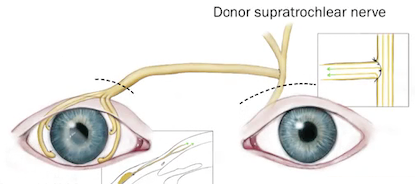


Figure1: Interpositional nerve graft coapted to healthy donor nerve^27^

**Follow-ups:**

Follow up of all patients will be done at baseline, day 1, day7, 1 month, 3 months, 6 months, 9 months and 1 year postoperatively.

**Outcome Measures**: -

**Primary outcomes**

1. To study the improvement in BCVA

2. To measure Quantitative improvement in corneal sensation by Cochet bonnet anesthesiometry in all quadrants of the cornea.

3. To observe the clarity of cornea as classified by Mackie in neurotrophic keratopathy

4. To calculate the increase in corneal subbasal nerve fiber plexus in terms of Subbasal nerve fiber length and sub-basal nerve fiber density evaluated on confocal microscopy.

5. The post-operative results will be compared with the non-affected (normal) eye to conclude how much normalization has been achieved by performing the procedure.

**Secondary outcomes**

To study any complications related to the intervention or surgical procedure in the recruited eyes.

**ETHICAL JUSTIFICATION:**

The proposed study aims at evaluating the efficacy of corneal neurotization using sural nerve graft coaptation to the contralateral supratrochlear nerve in patients with unilateral neurotrophic keratopathy and corneal anesthesia.

According to the guideline set up by ICMR (2000) and the Declaration of Helsinki (Modified 2008), the following will be adhered to in all patients/volunteers involved in the study:

1. The proposed study aims at evaluating the efficacy of corneal neurotization using contralateral supratrochlear nerve in patients with unilateral neurotrophic keratopathy and corneal anesthesia. The study will involve a complete ocular examination, clinical photography, routine blood, and confocal microscopy routinely followed by surgical corneal neurotisation procedure and do not pose any undue risks to the patient.

2. The patient will be explained about the motive of the study and the effect of the actual intervention in the language best understood by him/her. This study involves studying the clinical outcomes of cases following corneal neurotization.

3. Investigator will maintain the confidentiality of patients included in the study. Informed consent will be taken from all patients included in the study. The study will be in ethical boundaries and the appropriate management of patients will not be affected. Established guidelines as per the literature will be followed throughout the study.

4. The procedure will be recommended to the patients with visually significant NK showing no response to medical treatment.

5. Patients stay in hospital is not prolonged because of the study and patients will not be financially burdened at any point in the research. All efforts will be made to ensure that no extra visits are required for the study all the possible treatment options will be given and none will be withheld.

6. Written informed consent will be obtained from all prospective patients included in the study after informing them about the aims and methods of the study and the institutional affiliation of the researcher.

7. In publication of the results of this study, all efforts would be made to preserve the accuracy of both the positive and negative results of this study.

**STATISTICAL ANALYSIS**

• The analysis will be done with the help of ©SPSS version 25 for Windows (IBM Inc., Chicago, IL, USA). Data entries will be performed in pre-designed forms and excel sheets using ©Microsoft Excel for Windows.

• All the continuous variables will be checked for their normality using the Kolmogorov– Smirnov test.

∙To look for significant differences over the repeated measurements t-test (paired) will be used, in case of skewed data non-parametric test of Wilcoxon will be used.

• The data will be presented with descriptive statistics with Mean± SD or Median and Inter-quartile range as also their minimum and maximum values.

• For Categorical / Classified data, will be analyzed using the Chi-Square test or Fisher’s exact test, whichever is applicable. The data will be presented as frequencies, percentages, rates, etc.

• To observe the treatment effect, a t-test(Paired) or Wilcoxon signed-rank test will be applied depending upon whether the data is normality distributed or skewed respectively.

• p-value <0.05 will be considered statistically significant.

**INFORMATION TO PARTICIPANTS**

PROTOCOL NO:

SPONSOR: None

**INVESTIGATOR**: DR. AMAN KALIA

**GUIDE**: DR. MANU SAINI

NAME OF PARTICIPANT: …………………………………………………………………..

TITLE: “Clinical Outcomes of Corneal Neurotization using Sural Nerve Graft in Neurotrophic Keratopathy”

You are invited to take part in this research study. The information in this document is meant to help you decide whether or not to take part. Please feel free to ask if you have any queries or concerns. You are being asked to participate in this study being conducted in PGIMER, Chandigarh because you satisfy our eligibility criteria. You will be one of the patients we plan to recruit in this study.

**Purpose of research**

In the present study, we plan to evaluate the efficacy of corneal neurotization using contralateral supratrochlear nerve graft in patients with unilateral neurotrophic keratopathy and corneal anesthesia.

Information obtained from this study would be beneficial to other patients with the same disease.

We have obtained permission from the Institutional Ethics Committee [and the Drug Controller General of India] for conducting this study.

**Study Procedures**

The study involves baseline BCVA, Schirmer’s test 1, Fluorescein tear break up time (FTBUT), Ocular surface staining, Confocal Microscopy & Corneal sensations before the surgery followed by same investigations repeated post the surgery.

You may have to come to the hospital (study site) for examination and investigations apart from your scheduled visits if required.

**Possible benefits to you**

Treatment of your disease

**Possible benefits to other people**

The results of the research may provide benefits to society in terms of the advancement of medical knowledge and/or therapeutic benefit to future patients.

**The alternatives you have**

If you do not wish to participate, you have the alternative of getting the standard treatment for your condition.

**Cost to the participant**

You will not be paid to participate in this research study.

**Who is paying for this research?**

This is a thesis study and is not sponsored.

**What should you do in case of injury or a medical problem during this research study?**

Your safety is the prime concern of the research. If you are injured or have a medical problem as a result of being in this study, you should contact one of the people listed at the end of the consent form. You will be provided the required care/treatment.

**How long will the patient be admitted to the hospital for the procedure?**

The patient will be admitted 1-2 days before scheduled surgery to undergo necessary ocular investigations and pre-anesthesia checkups. The patient will be discharged on the next postoperative day after follow up examinations and will be called on scheduled follow up visits

**What complications can arise from this Procedure?**

Complications which may arise from the procedure are-

**A**. Early swelling around the eye, usually takes 10-15 days to resolve.

**B**. Numbness in the skin area from which the sural nerve will be harvested. It will take 3-6 months to improve and return to normal in most of the cases^37^.

**C**. Poor re-innervation of the affected cornea- If this would happen then there would be no discernible change in corneal sensation from pre-operative.

**D**. Additional minor risk of bleeding, infection, and poor scarring.

**Confidentiality of the information obtained from you**

You have the right to confidentiality regarding the privacy of your medical information (personal details, results of physical examinations, investigations, and your medical history).

By signing this document, you will be allowing the research team investigators, other study personnel, institutional ethics committee, and any person or agency required by law like the Drug Controller General of India to view your data, if required.

The results of clinical tests and therapy performed as part of this research may be included in your medical record. The information from this study, if published in scientific journals or presented at scientific meetings, will not reveal your identity.

**How will your decision to not participate in the study affect you?**

Your decision not to participate in this research study will not affect your medical care or your relationship with the investigator or the institution. Your doctor will still take care of you and you will not lose any benefits to which you are entitled.

**Can you decide to stop participating in the study once you start?**

Participation in this research is purely voluntary and you have the right to withdraw from this study at any time during the study without giving any reasons. However, it is advisable that you talk to the research team before withdrawing from the study.

**Can the investigator take you off the study?**

You may be taken off the study without your consent if you do not follow the instructions of the investigators or the research team or if the investigator thinks that further participation may cause you harm.

**Right to new information**

If the research team gets any new information during this research study that may affect your decision to continue participating in the study or may raise some doubts, you will be told about that information.

**Contact Persons**

For further information/questions, you can contact us at the following address:

Principal Investigator: Dr. Aman Kalia

Junior Resident,

Department of Ophthalmology,

PGIMER Chandigarh

9417861764

Guide: Dr. Manu Saini

Asst. Professor,

Department of Ophthalmology,

PGIMER Chandigarh

9999249626

In case of conflicts, you can contact the chairperson (convener) of our institutional ethics committee at the following address:

Dr ..... ... ... ... ... ... ... ... ... ... ..

Department of ... ... ... ... ... ... ... .

Convener/Chairperson,

Institutional Ethics Committee PGIMER, Chandigarh

Telephone:... ... ... ... ... . Fax:

**भागीदारों को जानकारी**

प्रोटोकॉल नंबर:

प्रायोजक: कोई नहीं

निवेशक: डॉ। अमन कालिया

गाइड: डॉ। मनु सैनी

प्रतिभागी का नाम: …………………………………………………………………..

शीर्षक: **एकतरफा न्यूरोट्रोपिक केराटोपोपैथी और कॉर्नियल एनेस्थीसिया वाले रोगियों में सुरल नर्व ग्राफ्ट को प्रतिपक्षी सुप्राट्रोक्लीअर तंत्रिका ग्राफ्ट** **के साथ जोड़ कर कॉर्निया न्यूरोटाइजेशन की प्रभावकारिता का मूल्यांकन करनI** I

आपको इस शोध अध्ययन में भाग लेने के लिए आमंत्रित किया गया है। इस दस्तावेज़ में जो जानकारी है यह तय करने में मदद करेगी कि आपको हिस्सा लेना है या नहीं। यदि आपके कोई प्रश्न या चिंता हैं, तो कृपया बेझिझक पूछें । आपको पीजीआईएमईआर, चंडीगढ़ में आयोजित किए जा रहे इस अध्ययन में भाग लेने के लिए कहा जा रहा है, क्योंकि आप हमारी पात्रता मानदंड को पूरा करते हैं। आप इस अध्ययन में भर्ती करने वाले मरीजों में से एक होंगे।

**अनुसंधान का उद्देश्य**

वर्तमान अध्ययन में, हम एकतरफा न्यूरोट्रोपिक केराटोपोपैथी और कॉर्नियल एनेस्थीसिया वाले रोगियों में प्रतिपक्षी सुपरट्रोचलेअर तंत्रिका ग्राफ्ट का उपयोग करके कॉर्निया न्यूरोटाइजेशन की प्रभावकारिता का मूल्यांकन करेंगे । इस अध्ययन से प्राप्त जानकारी उसी रोग के अन्य रोगियों के लिए फायदेमंद होगी।

हमने इस अध्ययन के संचालन के लिए संस्थागत आचार समिति [और भारत के ड्रग कंट्रोलर जनरल] से अनुमति प्राप्त की है।

**अध्ययन की प्रक्रियाएं**

अध्ययन में बेसलाइन बीसीवीए, शिमर का परीक्षण 1, फ्लूरोरेसेन का समय समाप्त हो जाना (FTBUT),ऑक्युलर सरफेस स्टैनिंग , कनफोकल माइक्रोस्कोपी, और कॉर्निया संवेदनाएं की जांच

शामिल है, जिन्हें सर्जरी के बाद दोहराया जायेगा ।

यदि आवश्यक हो, तो आपको अपनी निर्धारित जांच की यात्राओं से अलग से जांच के लिए अस्पताल (अध्ययन स्थल) आना पड़ सकता है ।

**आपको संभावित लाभ**

अपनी बीमारी का इलाज

**अन्य लोगों को संभावित लाभ**

अनुसंधान के परिणाम समाज को उन्नति की दृष्टि से लाभ प्रदान कर सकते हैं ,भविष्य के रोगियों को चिकित्सा ज्ञान और / या चिकित्सीय लाभ मिल सकता है ।

**आपके पास जो विकल्प हैं**

यदि आप भाग नहीं लेना चाहते हैं, तो आपके पास मानक उपचार प्राप्त करने का विकल्प है।

**प्रतिभागी को लागत**

इस शोध अध्ययन में भाग लेने के लिए आपको भुगतान नहीं किया जाएगा।

**इस शोध के लिए कौन भुगतान कर रहा है?**

यह एक थीसिस अध्ययन है और प्रायोजित नहीं है।

**इस शोध अध्ययन के दौरान चोट या किसी मेडिकल समस्या के मामले में आपको क्या करना चाहिए?**

आपकी सुरक्षा अनुसंधान की प्रमुख चिंता है। यदि आप घायल हैं या आपको कोई मेडिकल समस्या है तो इस अध्ययन में होने के परिणामस्वरूप, आपको सहमति पत्र के अंत में सूचीबद्ध लोगों में से एक से संपर्क करना चाहिए। आपको आवश्यक देखभाल / उपचार प्रदान किया जाएगा।

**कब तक प्रक्रिया के लिए रोगी को अस्पताल में भर्ती किया जाएगा?**

रोगी को निर्धारित सर्जरी से 1-2 दिन पहले भर्ती कराया जाएगा ताकि आवश्यक ओकुलर जांच और प्री-एनेस्थीसिया जांच से गुजरना पड़े। अनुवर्ती परीक्षाओं के बाद रोगी को अगले पोस्टऑपरेटिव दिन छुट्टी दे दी जाएगी और निर्धारित अनुवर्ती यात्राओं पर बुलाया जाएगा I

**इस प्रक्रिया से क्या जटिलताएँ पैदा हो सकती हैं?**

प्रक्रिया से उत्पन्न होने वाली जटिलताएं हैं-

A. आंख के आसपास शुरुआती सूजन, आमतौर पर हल करने में 10-15 दिन लगते हैं।

B. त्वचा के क्षेत्र में सुन्नता जिसमें से तंत्रिका तंत्रिका काटा जाएगा। अधिकांश मामलों में सुधार करने और सामान्य स्थिति में लौटने में 3-6 महीने लगेंगे।

ग। प्रभावित कॉर्निया का पुन: खराब होना- यदि ऐसा होता है तो प्री-ऑपरेटिव से कॉर्नियल सनसनी में कोई परिवर्तन नहीं होगा।

घ। रक्तस्राव, संक्रमण और खराब निशान का अतिरिक्त मामूली जोखिम।

**आपसे प्राप्त जानकारी की गोपनीयता**

आपको अपनी मेडिकल जानकारी की गोपनीयता के बारे में पूरा अधिकार है

(व्यक्तिगत विवरण, शारीरिक परीक्षाओं के परिणाम, जांच और आपका मेडिकल इतिहास)।

इस दस्तावेज़ पर हस्ताक्षर करके, यदि आवश्यक हो, तो आप अनुसंधान टीम के जांचकर्ताओं, अन्य अध्ययन के कर्मियों, संस्थागत नैतिकता समिति और कानून द्वारा आवश्यक किसी व्यक्ति या एजेंसी भारत के ड्रग कंट्रोलर जनरल की तरह कोअनुमति देंगे अपना डेटा देखने के लिए।

इस शोध के भाग के रूप में किए गए नैदानिक ​​परीक्षणों और चिकित्सा के परिणामों को आपके मेडिकल रिकॉर्ड में शामिल किया जा सकता हैI इस अध्ययन की जानकारी, यदि वैज्ञानिक पत्रिकाओं में प्रकाशित हुई या वैज्ञानिक बैठकों में प्रस्तुत हुई, आपकी पहचान को उजागर नहीं करेगा।

**अध्ययन में भाग नहीं लेने का आपका निर्णय आपको कैसे प्रभावित करेगा?**

इस शोध अध्ययन में भाग न लेने का आपका निर्णय आपकी चिकित्सा देखभाल को प्रभावित नहीं करेगा ना ही इस संस्था के साथ आपके सबंधों को । आपका डॉक्टर अभी भी आपका ध्यान रखेगाऔर आप कोई लाभ नहीं खोएंगे जिसके आप हकदार हैं।

**क्या आप शुरू करने से बाद अध्ययन में भाग लेना बंद करने का निर्णय ले सकते हैं?**

इस शोध में भागीदारी विशुद्ध रूप से स्वैच्छिक है और आपको बिना किसी कारण के अध्ययन के दौरान किसी भी समय इससे हटने का अधिकार है। हालांकि यह सलाह दी जाती है कि आप अध्ययन से हटने से पहले शोध टीम से बात करें।

**क्या जांचकर्ता आपको अध्ययन से दूर कर सकता है?**

यदि आप निर्देशों का पालन नहीं करते हैं या यदि जांचकर्ताओं या अनुसंधान दल या अन्वेषक को लगता है कि आगे की भागीदारी से आपको नुकसान पहुंचाता है तो आपकी सहमति के बिना आपको अध्ययन से हटा दिया जा सकता हैI

**नई जानकारी का अधिकार**

यदि इस शोध अध्ययन के दौरान शोध टीम को कोई नई जानकारी मिलती है जो आपके अध्ययन में भाग लेते रहने के निर्णय को प्रभावित कर सकती है, या कुछ संदेह उठा सकती है तो उस जानकारी के बारे में आपको बताया जाएगा।

**संपर्क करें**

अधिक जानकारी / प्रश्नों के लिए, आप हमें निम्नलिखित पते पर संपर्क कर सकते हैं:

प्रधान अन्वेषक: डॉ। अमन कालिया

जूनियर रेजिडेंट,

नेत्र रोग विभाग,

पीजीआईएमईआर, चंडीगढ़

9417861764

गाइड: डॉ। मनु सैनी

सहायक प्रोफेसर,

नेत्र रोग विभाग,

पीजीआईएमईआर, चंडीगढ़

9999249626

संघर्षों के मामले में, आप हमारे संस्थागत नैतिकता समिति के अध्यक्ष (संयोजक) से निम्नलिखित पते पर संपर्क कर सकते हैं:

डॉ ..... ..... ... ... ... ... ... ... ... ..

विभाग ... ... ... ... ... ... ... ।

संयोजक / अध्यक्ष,

संस्थागत आचार समिति पीजीआईएमईआर, चंडीगढ़

टेलीफोन: ... ... ... ... ...। फैक्स:.....................I

**ਭਾਗੀਦਾਰਾਂ ਨੂੰ ਜਾਣਕਾਰੀ**

ਪ੍ਰੋਟੋਕੋਲ ਨੰ:

ਸਪਾਂਸਰ: ਕੋਈ ਨਹੀਂ

ਖੋਜਕਾਰ: ਡਾ. ਅਮਨ ਕਾਲੀਆ

ਗਾਈਡ: ਡਾ. ਮੈਨੂ ਸੈਣੀ

ਭਾਗੀਦਾਰ ਦਾ ਨਾਮ: ……………………………………………………………… ..

ਟਾਈਟਲ: “**ਇਕਤਰਫਾ ਨਿਊਰੋਟਰੋਫਿਕ ਕੇਰਾਟੋਪੈਥੀ ਅਤੇ ਕੋਰਨੀਅਲ ਅਨੱਸਥੀਸੀਆ ਵਾਲੇ ਮਰੀਜ਼ਾਂ ਵਿੱਚ ਸੂਰਲ ਨਰਵ ਗ੍ਰਾਫਟ ਨੂੰ ਕੰਤ੍ਰਲਾਤੇਰਾਲ ਸੁਪ੍ਰੇਟ੍ਰੋਕਲੇਰ ਨਰਵ ਦੇ ਨਾਲ ਜੋੜ ਕੇ ਕਾਰਨੀਅਲ ਨਿਯੂਰੋਟਾਈਜ਼ੇਸ਼ਨ ਦੀ ਪ੍ਰਭਾਵਸ਼ੀਲਤਾ ਦਾ ਮੁਲਾਂਕਣ**”I

ਤੁਹਾਨੂੰ ਇਸ ਖੋਜ ਅਧਿਐਨ ਵਿਚ ਹਿੱਸਾ ਲੈਣ ਲਈ ਸੱਦਾ ਦਿੱਤਾ ਗਿਆ ਹI I ਇਸ ਦਸਤਾਵੇਜ਼ ਵਿਚ ਜਾਣਕਾਰੀ ਇਹ ਫੈਸਲਾ ਕਰਨ ਵਿਚ ਤੁਹਾਡੀ ਮਦਦ ਕਰੇਗੀ ਕਿ ਤੁਸੀਂ ਹਿੱਸਾ ਲੈਣਾ ਜਾਂ ਨਾ ਲੈਣਾ ਹੈ I ਕਿਰਪਾ ਕਰਕੇ ਇਹ ਪੁੱਛਣ ਲਈ ਸੁਤੰਤਰ ਮਹਿਸੂਸ ਕਰੋ ਜੇ ਕਰ ਤੁਹਾਡੇ ਕੋਲ ਕੋਈ ਪ੍ਰਸ਼ਨ ਹਨ ਜਾਂ ਚਿੰਤਾਵਾਂ ਹਨI ਤੁਹਾਨੂੰ ਪੀਜੀਆਈਐਮਈਆਰ ,ਚੰਡੀਗੜ੍ਹ ਵਿੱਚ ਕਰਵਾਏ ਜਾ ਰਹੇ ਇਸ ਅਧਿਐਨ ਵਿੱਚ ਹਿੱਸਾ ਲੈਣ ਲਈ ਕਿਹਾ ਜਾ ਰਿਹਾ ਹੈ, ਕਿਉਂਕਿ ਤੁਸੀਂ ਸਾਡੇ ਯੋਗਤਾ ਦੇ ਮਾਪਦੰਡਾਂ ਨੂੰ ਪੂਰਾ ਕਰਦੇ ਹੋ I ਤੁਸੀਂ ਇਸ ਅਧਿਐਨ ਵਿਚ ਭਰਤੀ ਕੀਤੇ ਜਾਣ ਵਾਲੇ ਮਰੀਜ਼ਾਂ ਵਿਚੋਂ ਇਕ ਹੋਵੋਗੇ I

**ਖੋਜ ਦਾ ਉਦੇਸ਼**

ਮੌਜੂਦਾ ਅਧਿਐਨ ਵਿੱਚ, ਅਸੀਂ ਇਕਤਰਫਾ ਨਿਊਰੋਟਰੋਫਿਕ ਕੇਰਾਟੋਪੈਥੀ ਅਤੇ ਕੋਰਨੀਅਲ ਅਨੱਸਥੀਸੀਆ ਵਾਲੇ ਮਰੀਜ਼ਾਂ ਵਿੱਚ ਕੰਤ੍ਰਲਾਤੇਰਾਲ ਸੁਪ੍ਰੇਟ੍ਰੋਕਲੇਰ ਨਰਵ ਦੀ ਵਰਤੋਂ ਕਰਕੇ ਕਾਰਨੀਅਲ ਨਿਯੂਰੋਟਾਈਜ਼ੇਸ਼ਨ ਦੀ ਪ੍ਰਭਾਵਸ਼ੀਲਤਾ ਦਾ ਮੁਲਾਂਕਣ ਕਾਰਾਂਗੇ Iਇਸ ਅਧਿਐਨ ਤੋਂ ਪ੍ਰਾਪਤ ਕੀਤੀ ਜਾਣਕਾਰੀ ਇਸ ਬਿਮਾਰੀ ਦੇ ਦੂਜੇ ਮਰੀਜ਼ਾਂ ਲਈ ਵੀ ਲਾਭਦਾਇਕ ਹੋਵੇਗੀ I

ਅਸੀਂ ਸੰਸਥਾਗਤ ਨੈਤਿਕਤਾ ਕਮੇਟੀ [ਅਤੇ ਡਰੱਗ ਕੰਟਰੋਲਰ ਜਨਰਲ ਆਫ਼ ਇੰਡੀਆ] ਤੋਂ ਇਸ ਅਧਿਐਨ ਨੂੰ ਕਰਵਾਉਣ ਲਈ ਆਗਿਆ ਪ੍ਰਾਪਤ ਕੀਤੀ ਹੈ I

**ਅਧਿਐਨ ਪ੍ਰਕਿਰਿਆਵਾਂ**

ਅਧਿਐਨ ਵਿੱਚ ਬੇਸਲਾਈਨ ਬੀਸੀਵੀਏ, ਸ਼ੀਰਮਰ ਦਾ ਟੈਸਟ 1, ਫਲੋਰੋਸਿਨ ਟੀਅਰ ਬਰੇਕ ਅਪ ਟਾਈਮ (ਐਫ.ਟੀ.ਬੀ.ਯੂ.ਟੀ.), ਓਕੂਲਰ ਸਤਹ ਸਟਾਈਨਿੰਗ, ਕਨਫੋਕਲ ਮਾਈਕਰੋਸਕੋਪੀ ਅਤੇ ਕੋਰਨੀਅਲ ਸਨਸਨੀ ਟੈਸਟ ਸ਼ਾਮਲ ਹਨ ਜੋ ਸਰਜਰੀ ਤੋਂ ਬਾਅਦ ਦੁਹਰਾਏ ਜਾਣਗੇ.Iਹੋ ਸਕਦਾ ਹੈ ਕਿ ਜੇ ਜਰੂਰੀ ਹੋਵੇ, ਤਾਂ ਤੁਹਾਨੂੰ ਹਸਪਤਾਲ (ਅਧਿਐਨ ਵਾਲੀ ਥਾਂ) ਤੇ ਤੁਹਾਡੀਆਂ ਤਹਿ ਕੀਤੀਆਂ ਜਾਂਚ ਲਈ ਮੁਲਾਕਾਤਾਂ ਤੋਂ ਇਲਾਵਾ ਜਾਂਚ ਲਈ ਆਉਣਾ ਪਵੇI

**ਤੁਹਾਡੇ ਲਈ ਸੰਭਵ ਲਾਭ**

ਤੁਹਾਡੀ ਬਿਮਾਰੀ ਦਾ ਇਲਾਜ I

**ਦੂਜੇ ਲੋਕਾਂ ਨੂੰ ਸੰਭਾਵਤ ਲਾਭ**

ਖੋਜ ਦੇ ਨਤੀਜੇ ਸਮਾਜ ਨੂੰ ਅੱਗੇ ਵਧਾਉਣ ਦੇ ਮਾਮਲੇ ਵਿਚ ਲਾਭ ਪ੍ਰਦਾਨ ਕਰ ਸਕਦੇ ਹਨ ਮੈਡੀਕਲ ਗਿਆਨ ਜਾਂ ਭਵਿੱਖ ਦੇ ਮਰੀਜ਼ਾਂ ਲਈ ਇਲਾਜ ਲਾਭ ਦੇ ਰੂਪ ਵਿੱਚ I

**ਤੁਹਾਡੇ ਕੋਲ ਵਿਕਲਪ**

ਜੇ ਤੁਸੀਂ ਹਿੱਸਾ ਲੈਣਾ ਨਹੀਂ ਚਾਹੁੰਦੇ ਹੋ, ਤਾਂ ਤੁਹਾਡੇ ਕੋਲ ਮਿਆਰੀ ਇਲਾਜ ਕਰਵਾਉਣ ਦਾ ਵਿਕਲਪ ਹੈ I

**ਭਾਗੀਦਾਰ ਨੂੰ ਲਾਗਤ**

ਇਸ ਖੋਜ ਅਧਿਐਨ ਵਿਚ ਹਿੱਸਾ ਲੈਣ ਲਈ ਤੁਹਾਨੂੰ ਭੁਗਤਾਨ ਨਹੀਂ ਕੀਤਾ ਜਾਵੇਗਾ I

**ਇਸ ਖੋਜ ਲਈ ਕੌਣ ਭੁਗਤਾਨ ਕਰ ਰਿਹਾ ਹੈ ?**

ਇਹ ਇਕ ਥੀਸਸ ਅਧਿਐਨ ਹੈ ਅਤੇ ਇਸ ਨੂੰ ਪ੍ਰਯੋਜਿਤ ਨਹੀਂ ਕੀਤਾ ਗਿਆ ਹੈ I

**ਇਸ ਖੋਜ ਅਧਿਐਨ ਦੇ ਦੌਰਾਨ ਸੱਟ ਜਾਂ ਡਾਕਟਰੀ ਸਮੱਸਿਆ ਦੇ ਮਾਮਲੇ ਵਿੱਚ ਤੁਹਾਨੂੰ ਕੀ ਕਰਨਾ ਚਾਹੀਦਾ ਹੈ ?**

ਤੁਹਾਡੀ ਸੁਰੱਖਿਆ ਖੋਜ ਦੀ ਮੁੱਖ ਚਿੰਤਾ ਹੈ. ਜੇ ਤੁਸੀਂ ਇਸ ਅਧਿਐਨ ਵਿੱਚ ਸ਼ਾਮਲ ਹੋਣ ਦੇ ਨਤੀਜੇ ਵਜੋਂ ਜ਼ਖਮੀ ਹੋ ਜਾਂ ਕੋਈ ਡਾਕਟਰੀ ਸਮੱਸਿਆ ਹੈ, ਤਾਂ ਤੁਹਾਨੂੰ ਸਹਿਮਤੀ ਫਾਰਮ ਦੇ ਅੰਤ ਵਿੱਚ ਸੂਚੀਬੱਧ ਲੋਕਾਂ ਵਿੱਚੋਂ ਇੱਕ ਨਾਲ ਸੰਪਰਕ ਕਰਨਾ ਚਾਹੀਦਾ ਹੈ I ਤੁਹਾਨੂੰ ਲੋੜੀਂਦੀ ਦੇਖਭਾਲ / ਇਲਾਜ ਪ੍ਰਦਾਨ ਕੀਤਾ ਜਾਵੇਗਾI

**ਇਸ ਵਿਧੀ ਲਈ ਮਰੀਜ਼ ਨੂੰ ਕਿੰਨਾ ਚਿਰ ਹਸਪਤਾਲ ਵਿੱਚ ਦਾਖਲ ਕੀਤਾ ਜਾਵੇਗਾ?**

ਮਰੀਜ਼ ਨੂੰ ਨਿਯਮਤ ਸਰਜਰੀ ਤੋਂ 1-2 ਦਿਨ ਪਹਿਲਾਂ ਦਾਖਲ ਕਰਵਾਇਆ ਜਾਏਗਾ ਤਾਂ ਜੋ ਜ਼ਰੂਰੀ ਅੱਖਾਂ ਦੀ ਜਾਂਚ ਅਤੇ ਪ੍ਰੀ-ਅਨੱਸਥੀਸੀਆ ਜਾਂਚ ਕੀਤੀ ਜਾ ਸਕੇ. ਫਾਲੋ-ਅਪ ਪ੍ਰੀਖਿਆਵਾਂ ਦੇ ਬਾਅਦ ਅਗਲੇ ਪੋਸਟੋਪਰੇਟਿਵ ਦਿਨ ਮਰੀਜ਼ ਨੂੰ ਛੁੱਟੀ ਦੇ ਦਿੱਤੀ ਜਾਏਗੀ ਅਤੇ ਨਿਰਧਾਰਤ ਫਾਲੋ-ਅਪ ਮੁਲਾਕਾਤਾਂ ਤੇ ਬੁਲਾਇਆ ਜਾਵੇਗਾI

**ਇਸ ਪ੍ਰਕ੍ਰਿਆ ਵਿਚੋਂ ਕਿਹੜੀਆਂ ਪੇਚੀਦਗੀਆਂ ਪੈਦਾ ਹੋ ਸਕਦੀਆਂ ਹਨ?**

ਪੇਚੀਦਗੀਆਂ ਜਿਹੜੀਆਂ ਪ੍ਰਕ੍ਰਿਆ ਤੋਂ ਪੈਦਾ ਹੋ ਸਕਦੀਆਂ ਹਨ -

ਏ. ਅੱਖ ਦੇ ਦੁਆਲੇ ਜਲਦੀ ਸੋਜ ਹੋਣਾ, ਆਮ ਤੌਰ 'ਤੇ ਠੀਕ ਹੋਣ ਲਈ 10-15 ਦਿਨ ਲੈਂਦਾ ਹੈI

ਬੀ. ਚਮੜੀ ਦੇ ਉਸ ਖੇਤਰ ਵਿਚ ਸੁੰਨਤਾ ਜਿਸ ਤੋਂ ਸੂਰਲ ਨਸ ਦੀ ਕਟਾਈ ਕੀਤੀ ਜਾਏਗੀ. ਜ਼ਿਆਦਾਤਰ ਮਾਮਲਿਆਂ ਵਿੱਚ ਸੁਧਾਰ ਕਰਨ ਅਤੇ ਆਮ ਵਾਂਗ ਵਾਪਸੀ ਵਿੱਚ 3-6 ਮਹੀਨੇ ਲੱਗਣਗੇI

ਸੀ. ਪ੍ਰਭਾਵਿਤ ਕੌਰਨੀਆ ਦੀ ਮਾੜੀ ਪੁਨਰ-ਪ੍ਰਵਾਹ, ਜੇ ਅਜਿਹਾ ਹੁੰਦਾ ਤਾਂ ਪ੍ਰੀ-ਆਪਰੇਟਿਵ ਤੋਂ ਕਾਰਨੀਅਲ ਸਨਸਨੀ ਵਿਚ ਕੋਈ ਸਪਸ਼ਟ ਤਬਦੀਲੀ ਨਹੀਂ ਹੁੰਦੀI

ਡੀ. ਖੂਨ ਵਗਣਾ, ਸੰਕਰਮਣ ਅਤੇ ਮਾੜੇ ਦਾਗ ਹੋਣ ਦੇ ਵਾਧੂ ਮਾਮੂਲੀ ਜੋਖਮI

**ਤੁਹਾਡੇ ਤੋਂ ਪ੍ਰਾਪਤ ਕੀਤੀ ਜਾਣਕਾਰੀ ਦੀ ਗੁਪਤਤਾ**

ਤੁਹਾਡੀ ਡਾਕਟਰੀ ਜਾਣਕਾਰੀ ਦੀ ਨਿੱਜਤਾ ਦੇ ਸੰਬੰਧ ਵਿੱਚ ਤੁਹਾਨੂੰ ਗੁਪਤਤਾ ਪ੍ਰਾਪਤ ਕਰਨ ਦਾ ਅਧਿਕਾਰ ਹੈ

(ਨਿੱਜੀ ਵੇਰਵੇ, ਸਰੀਰਕ ਮੁਆਇਨਾ ਦੇ ਨਤੀਜੇ, ਪੜਤਾਲ ਅਤੇ ਤੁਹਾਡੇ ਡਾਕਟਰੀ ਇਤਿਹਾਸ).

ਇਸ ਦਸਤਾਵੇਜ਼ ਤੇ ਦਸਤਖਤ ਕਰਨ ਨਾਲ, ਤੁਸੀਂ ਖੋਜ ਟੀਮ ਦੇ ਜਾਂਚਕਰਤਾਵਾਂ, ਹੋਰ ਅਧਿਐਨ ਕਰਮਚਾਰੀ , ਸੰਸਥਾਗਤ ਨੈਤਿਕਤਾ ਕਮੇਟੀ ਅਤੇ ਕਾਨੂੰਨ ਦੁਆਰਾ ਲੋੜੀਂਦਾ ਕੋਈ ਵੀ ਵਿਅਕਤੀ ਜਾਂ ਏਜੰਸੀ ਭਾਰਤ ਦੇ ਡਰੱਗ ਕੰਟਰੋਲਰ ਜਨਰਲ ਵਾਂਗ ਤੁਹਾਡੇ ਡੇਟਾ ਨੂੰ ਵੇਖਣ ਲਈ, ਜੇ ਜਰੂਰੀ ਹੋਵੇ ,ਦੀ ਆਗਿਆ ਦੇਵੋਗੇ I

ਇਸ ਖੋਜ ਦੇ ਹਿੱਸੇ ਵਜੋਂ ਕੀਤੇ ਗਏ ਕਲੀਨਿਕਲ ਟੈਸਟਾਂ ਅਤੇ ਥੈਰੇਪੀ ਦੇ ਨਤੀਜੇ ਤੁਹਾਡੇ ਡਾਕਟਰੀ ਰਿਕਾਰਡ ਵਿੱਚ ਸ਼ਾਮਲ ਕੀਤੇ ਜਾ ਸਕਦੇ ਹਨ I ਇਸ ਅਧਿਐਨ ਤੋਂ ਜਾਣਕਾਰੀ, ਜੇ ਕਰ ਵਿਗਿਆਨਕ ਰਸਾਲਿਆਂ ਵਿੱਚ ਪ੍ਰਕਾਸ਼ਤ ਕੀਤੀ ਗਈ ਜਾਂ ਵਿਗਿਆਨਕ ਮੀਟਿੰਗਾਂ ਵਿਚ ਪੇਸ਼ ਕੀਤੀ ਗਈ, ਤੁਹਾਡੀ ਪਛਾਣ ਨਹੀਂ ਜ਼ਾਹਰ ਕਰੇਗਾ I

**ਅਧਿਐਨ ਵਿਚ ਹਿੱਸਾ ਨਾ ਲੈਣ ਦੇ ਤੁਹਾਡੇ ਫੈਸਲੇ ਦਾ ਤੁਹਾਡੇ 'ਤੇ ਕੀ ਅਸਰ ਪਏਗਾ ?**

ਇਸ ਖੋਜ ਅਧਿਐਨ ਵਿਚ ਹਿੱਸਾ ਨਾ ਲੈਣ ਦੇ ਤੁਹਾਡੇ ਫੈਸਲੇ ਦਾ ਤੁਹਾਡੀ ਡਾਕਟਰੀ ਦੇਖਭਾਲ 'ਤੇ ਕੋਈ ਅਸਰ ਨਹੀਂ ਪਏਗਾ ਨਾਂ ਹੀ ਜਾਂਚਕਰਤਾ ਜਾਂ ਸੰਸਥਾ ਨਾਲ ਤੁਹਾਡੇ ਸੰਬੰਧ ਤੇ I ਤੁਹਾਡਾ ਡਾਕਟਰ ਫਿਰ ਵੀ ਤੁਹਾਡੀ ਦੇਖਭਾਲ ਕਰੇਗਾ I ਅਤੇ ਤੁਸੀਂ ਕੋਈ ਲਾਭ ਨਹੀਂ ਗੁਆਓਗੇ ਜਿਸ ਦੇ ਤੁਸੀਂ ਹੱਕਦਾਰ ਹੋ I

**ਕੀ ਤੁਸੀਂ ਇਕ ਵਾਰ ਅਧਿਐਨ ਵਿਚ ਹਿੱਸਾ ਲੈਣਾ ਬੰਦ ਕਰਨ ਦਾ ਫੈਸਲਾ ਕਰ ਸਕਦੇ ਹੋ ?**

ਇਸ ਖੋਜ ਵਿਚ ਹਿੱਸਾ ਲੈਣਾ ਪੂਰੀ ਤਰ੍ਹਾਂ ਸਵੈ-ਇੱਛੁਕ ਹੈ ਅਤੇ ਤੁਹਾਨੂੰ ਇਸ ਤੋਂ ਪਿੱਛੇ ਹਟਣ ਦਾ ਅਧਿਕਾਰ ਹੈ

ਬਿਨਾਂ ਕਿਸੇ ਕਾਰਨ ਦੱਸੇ ਅਧਿਐਨ ਦੌਰਾਨ ਕਿਸੇ ਵੀ ਸਮੇਂ ਇਹ ਅਧਿਐਨ ਤੋਂ ਪਿੱਛੇ ਹਟ ਸਕਦੇ ਹੋ I ਪਰ, ਤੁਹਾਨੂੰ

ਸਲਾਹ ਦਿੱਤੀ ਜਾਂਦੀ ਹੈ ਕਿ ਤੁਸੀਂ ਅਧਿਐਨ ਤੋਂ ਪਿੱਛੇ ਹਟਣ ਤੋਂ ਪਹਿਲਾਂ ਖੋਜ ਟੀਮ ਨਾਲ ਗੱਲ ਕਰੋ I

**ਕੀ ਪੜਤਾਲ ਕਰਨ ਵਾਲਾ ਤੁਹਾਨੂੰ ਅਧਿਐਨ ਤੋਂ ਬਾਹਰ ਕਰ ਸਕਦਾ ਹੈ ?**

ਜੇ ਤੁਸੀਂ ਇਸ ਦੇ ਨਿਰਦੇਸ਼ਾਂ ਦੀ ਪਾਲਣਾ ਨਹੀਂ ਕਰਦੇ ਤਾਂ ਤੁਹਾਡੀ ਸਹਿਮਤੀ ਤੋਂ ਬਿਨਾਂ ਤੁਹਾਨੂੰ ਅਧਿਐਨ ਤੋਂ ਬਾਹਰ ਕੀਤਾ ਜਾ ਸਕਦਾ ਹੈ ਜਾਂ ਖੋਜ ਟੀਮ ਜਾਂ ਜੇ ਜਾਂਚਕਰਤਾ ਇਹ ਸੋਚਦਾ ਹੈ ਕਿ ਹੋਰ ਭਾਗੀਦਾਰੀ ਤੁਹਾਨੂੰ ਨੁਕਸਾਨ ਪਹੁੰਚਾਉਣ ਦਾ ਕਾਰਨ ਹੋ ਸਕਦੀ ਹੈ ਤਾਂ ਤੁਹਾਨੂੰ ਅਧਿਐਨ ਤੋਂ ਬਾਹਰ ਕੀਤਾ ਜਾ ਸਕਦਾ ਹੈ I

**ਨਵੀਂ ਜਾਣਕਾਰੀ ਦਾ ਅਧਿਕਾਰ**

ਜੇ ਖੋਜ ਟੀਮ ਇਸ ਖੋਜ ਅਧਿਐਨ ਦੌਰਾਨ ਕੋਈ ਨਵੀਂ ਜਾਣਕਾਰੀ ਪ੍ਰਾਪਤ ਕਰਦੀ ਹੈ ਜੋ ਤੁਹਾਡੇ ਅਧਿਐਨ ਵਿਚ ਹਿੱਸਾ ਜਾਰੀ ਰੱਖਣ ਦੇ ਫ਼ੈਸਲੇ 'ਤੇ ਅਸਰ ਪਾ ਸਕਦੀ ਹੈ, ਜਾਂ ਕੁਝ ਸ਼ੰਕੇ ਪੈਦਾ ਹੋ ਸਕਦੇ ਹਨ, ਤਾਂ ਉਸ ਜਾਣਕਾਰੀ ਬਾਰੇ ਤੁਹਾਨੂੰ ਦੱਸਿਆ ਜਾਵੇਗਾ I

**ਸੰਪਰਕ ਵਿਅਕਤੀ**

ਵਧੇਰੇ ਜਾਣਕਾਰੀ / ਪ੍ਰਸ਼ਨਾਂ ਲਈ, ਤੁਸੀਂ ਹੇਠ ਦਿੱਤੇ ਪਤੇ ਤੇ ਸਾਡੇ ਨਾਲ ਸੰਪਰਕ ਕਰ ਸਕਦੇ ਹੋ:

ਪ੍ਰਮੁੱਖ ਜਾਂਚਕਰਤਾ: ਡਾ:ਅਮਨ ਕਾਲੀਆ

ਜੂਨੀਅਰ ਰੇਸੀਡੇੰਟ,

ਨੇਤਰ ਵਿਗਿਆਨ ਵਿਭਾਗ,

ਪੀ.ਜੀ.ਐਮ.ਆਈ.ਆਰ. ਚੰਡੀਗੜ੍ਹ

9417861764

ਗਾਈਡ: ਡਾ: ਮਨੂ ਸੈਣੀ

ਸਹਾਇਕ ਪ੍ਰੋਫੈਸਰ,

ਨੇਤਰ ਵਿਗਿਆਨ ਵਿਭਾਗ,

ਪੀ.ਜੀ.ਐਮ.ਆਈ.ਆਰ. ਚੰਡੀਗੜ੍ਹ

9999249626

ਵਿਵਾਦਾਂ ਦੀ ਸਥਿਤੀ ਵਿੱਚ, ਤੁਸੀਂ ਸਾਡੀ ਸੰਸਥਾਗਤ ਨੈਤਿਕਤਾ ਕਮੇਟੀ ਦੇ ਚੇਅਰਪਰਸਨ (ਕਨਵੀਨਰ) ਨਾਲ ਹੇਠ

ਦਿੱਤੇ ਪਤੇ ਤੇ ਸੰਪਰਕ ਕਰ ਸਕਦੇ ਹੋ:

ਡਾ ..... ..... ... ... ... ... ... ... ... ... .. ..

ਵਿਭਾਗ ... ... ... ... ... ... ....

ਕਨਵੀਨਰ / ਚੇਅਰਪਰਸਨ,

ਇੰਸਟੀਚਿ .ਸ਼ਨਲ ਐਥਿਕਸ ਕਮੇਟੀ ਪੀ.ਜੀ.ਐੱਮ.ਈ.ਐੱਮ.ਆਰ, ਚੰਡੀਗੜ੍ਹ

ਟੈਲੀਫੋਨ: ... ... ... ... .... ਫੈਕਸ:......................

**PATIENT CONSENT FORM**

Protocol / Study number :______________________

Participant identification number for this trial: _______________________

**Title of project:** Clinical Outcomes of Corneal Neurotization using Sural Nerve Graft in Neurotrophic Keratopathy

Principal Investigator: Dr. Aman Kalia Ph: 9417861764

Chief Guide: Dr. Manu Saini Ph: 9999249626

The contents of the information sheet dated that was provided have been read carefully by me/explained in detail to me, in a language that I comprehend, and I have fully understood the contents. I confirm that I have had the opportunity to ask questions.

The nature and purpose of the study and its potential risks/benefits and expected duration of the study and other relevant details of the study have been explained to me in detail. I understand that my participation is voluntary and that I am free to withdraw at any time, without giving any reason, without my medical care or legal right being affected.

I understand that the information collected about me from my participation in this research and sections of any of my medical notes may be looked at by responsible individuals from PGIMER. I permit these individuals to have access to my records.

I agree to take part in the above study.

--------------------------------------------- Date:

(Signatures / Left Thumb Impression) Place:

Name of the Participant: ____________________________________

Son / Daughter / Spouse of:__________________________________

Complete postal address: _____________________________________

This is to certify that the above consent has been obtained in my presence.

------------------------------ Date:

Signature of the Principal Investigator Place:

1) Witness – 1 2) Witness – 2

------------------------------ --------------------------------

Signatures Signatures

Name: Name:

Address: Address:

**रोगी सहमति फार्म**

प्रोटोकॉल / अध्ययन संख्या: ______________________

इस परीक्षण के लिए प्रतिभागी की पहचान संख्या: _______________________

परियोजना का शीर्षक: **एकतरफा न्यूरोट्रोपिक केराटोपोपैथी और कॉर्नियल एनेस्थीसिया वाले रोगियों में प्रतिपक्षी सुप्राट्रोक्लीअर तंत्रिका ग्राफ्ट का उपयोग करके कॉर्निया न्यूरोटाइजेशन की प्रभावकारिता का मूल्यांकन** ।

प्रधान अन्वेषक: डॉ अमन कालिया Ph: 9417861764

मुख्य मार्गदर्शक: डॉ मनु सैनी Ph: 9999249626

उपलब्ध कराई गई सूचना पत्र की सामग्री को मेरे द्वारा ध्यान से पढ़ा गया है, मुझे विस्तार से उस भाषा में जिसे मैं समझतi हूं, समझाया गया है ,और मैंने पूरी तरह से सामग्री को समझा है । मैं पुष्टि करता हूं कि मुझे प्रश्न पूछने का अवसर मिला है।

अध्ययन की प्रकृति और उद्देश्य और इसके संभावित और अपेक्षित जोखिम / लाभ ,अध्ययन की अवधि, और अध्ययन के अन्य प्रासंगिक विवरणों को मुझे विस्तार से समझाया गया है। मैं समझता हूं कि मेरी भागीदारी स्वैच्छिक है और जिसे मैं किसी भी समय, बिना किसी कारण के, बिना मेरी चिकित्सा देखभाल या कानूनी अधिकार प्रभावित हुए वापस लेने के लिए स्वतंत्र हूं ।

मैं समझता हूं कि इसमें मेरी भागीदारी से मेरे बारे में जानकारी एकत्र की गई हैIमेरे किसी भी मेडिकल अनुसंधान के नोट और वर्गों को स्नातकोत्तर चिकित्सा शिक्षा एवं अनुसंधान संस्थान (पी॰जी॰आई॰एम॰ई॰आर॰) , चंडीगढ़ के जिम्मेदार व्यक्तियों द्वारा देखा जा सकता है। मैं इन व्यक्तियों को अपने रिकॉर्ड तक पहुंचने की अनुमति देता हूं।

मैं उपरोक्त अध्ययन में भाग लेने के लिए सहमत हूं।

--------------------------------------------- तारीख:

(हस्ताक्षर / बाएं अंगूठे का निशान) जगह:

प्रतिभागी का नाम: ____________________________________

पुत्र / पुत्री / पति / पत्नी: __________________________________

पूरा डाक पता: _____________________________________

यह प्रमाणित करना है कि मेरी उपस्थिति में उपरोक्त सहमति प्राप्त की गई है।

------------------------------ तारीख:

प्रधान अन्वेषक का हस्ताक्षर स्थान:

1) गवाह – 1 2) गवाह - 2

------------------------------ -------------------- ------------

हस्ताक्षर हस्ताक्षर

नाम नाम:

पता पता:

**ਮਰੀਜ਼ ਸਹਿਮਤੀ ਫਾਰਮ**

ਪ੍ਰੋਟੋਕੋਲ / ਅਧਿਐਨ ਨੰਬਰ: ______________________

ਇਸ ਅਜ਼ਮਾਇਸ਼ ਲਈ ਭਾਗੀਦਾਰ ਦੀ ਪਛਾਣ ਨੰਬਰ: _______________________

ਪ੍ਰੋਜੈਕਟ ਦਾ ਸਿਰਲੇਖ: **ਇਕਤਰਫਾ ਨਿਊਰੋਟਰੋਫਿਕ ਕੇਰਾਟੋਪੈਥੀ ਅਤੇ ਕੋਰਨੀਅਲ ਅਨੱਸਥੀਸੀਆ ਵਾਲੇ ਮਰੀਜ਼ਾਂ ਵਿੱਚ ਕੰਤ੍ਰਲਾਤੇਰਾਲ ਸੁਪ੍ਰੇਟ੍ਰੋਕਲੇਰ ਨਰਵ ਦੀ ਵਰਤੋਂ ਕਰਕੇ ਕਾਰਨੀਅਲ ਨਿਯੂਰੋਟਾਈਜ਼ੇਸ਼ਨ ਦੀ ਪ੍ਰਭਾਵਸ਼ੀਲਤਾ ਦਾ ਮੁਲਾਂਕਣ** I

ਪ੍ਰਮੁੱਖ ਜਾਂਚਕਰਤਾ: ਡਾ ਅਮਨ ਕਾਲੀਆ ਫੋਨ: 9417861764

ਮੁੱਖ ਗਾਈਡ: ਡਾ: ਮਨੂੰ ਸੈਣੀ ਫੋਨ: 9999249626

ਦਿੱਤੀ ਗਈ ਜਾਣਕਾਰੀ ਸ਼ੀਟ ਦੇ ਭਾਗਾਂ ਨੂੰ ਮੇਰੇ ਦੁਆਰਾ ਧਿਆਨ ਨਾਲ ਪੜ੍ਹਿਆ ਗਿਆ ਅਤੇ ਵਿਸਥਾਰ ਨਾਲ , ਜਿਸ ਭਾਸ਼ਾ ਵਿਚ ਮੈਂ ਸਮਝਦਾ ਹਾਂ ਸਮਝਾਇਆ ਗਿਆ, ਅਤੇ ਮੈਂ ਸਮਗਰੀ ਨੂੰ ਚੰਗੀ ਤਰ੍ਹਾਂ ਸਮਝ ਲਿਆ ਹੈI ਮੈਂ ਪੁਸ਼ਟੀ ਕਰਦਾ ਹਾਂ ਕਿ ਮੈਨੂੰ ਪ੍ਰਸ਼ਨ ਪੁੱਛਣ ਦਾ ਮੌਕਾ ਮਿਲਿਆ ਹੈ I

ਅਧਿਐਨ ਦਾ ਸੁਭਾਅ ਅਤੇ ਉਦੇਸ਼ ਅਤੇ ਇਸਦੇ ਸੰਭਾਵਿਤ ਜੋਖਮ / ਲਾਭ ਅਤੇ ਅਧਿਐਨ ਦੀ ਅਨੁਮਾਨਤ ਅਵਧੀ, ਅਤੇ ਅਧਿਐਨ ਦੇ ਹੋਰ ਉਚਿਤ ਕਵੇਂ ਵੇਰਵਿਆਂ ਬਾਰੇ ਮੈਨੂੰ ਵਿਸਥਾਰ ਨਾਲ ਦੱਸਿਆ ਗਿਆ ਹੈI ਮੈਂ ਸਮਝਦਾ / ਸਮਝਦੀ ਹਾਂ ਕਿ ਮੇਰੀ ਭਾਗੀਦਾਰੀ ਸਵੈਇੱਛਤ ਹੈ ਅਤੇ ਮੈਂ ਬਿਨਾਂ ਕਿਸੇ ਕਾਰਨ ਦੱਸੇ ਆਪਣੀ ਡਾਕਟਰੀ ਦੇਖਭਾਲ ਜਾਂ ਕਾਨੂੰਨੀ ਅਧਿਕਾਰ ਦੇ ਪ੍ਰਭਾਵਿਤ ਹੋਏ ਬਿਨਾਂ ਕਿਸੇ ਵੀ ਸਮੇਂ ਵਾਪਸ ਲੈਣ ਲਈ ਸੁਤੰਤਰ ਹਾਂ I

ਮੈਂ ਸਮਝਦਾ / ਸਮਝਦੀ ਹਾਂ ਕਿ ਇਸ ਖੋਜ ਵਿਚ ਮੇਰੀ ਭਾਗੀਦਾਰੀ ਤੋਂ ਮੇਰੇ ਬਾਰੇ ਇਕੱਠੀ ਕੀਤੀ ਗਈ ਜਾਣਕਾਰੀ ਅਤੇ ਮੇਰੇ ਕਿਸੇ ਵੀ ਮੈਡੀਕਲ ਨੋਟ ਦੇ ਭਾਗ ਪੀਜੀਆਈਐਮਈਆਰ ਦੇ ਜ਼ਿੰਮੇਵਾਰ ਵਿਅਕਤੀਆਂ ਦੁਆਰਾ ਦੇਖੇ ਜਾ ਸਕਦੇ ਹਨ I ਮੈਂ ਇਨ੍ਹਾਂ ਵਿਅਕਤੀਆਂ ਨੂੰ ਮੇਰੇ ਰਿਕਾਰਡਾਂ ਤਕ ਪਹੁੰਚ ਕਰਨ ਦੀ ਆਗਿਆ ਦਿੰਦਾ ਹਾਂ I

ਮੈਂ ਉਪਰੋਕਤ ਅਧਿਐਨ ਵਿਚ ਹਿੱਸਾ ਲੈਣ ਲਈ ਸਹਿਮਤ ਹਾਂ

. --------------------------------------------- ਤਾਰੀਖ਼:

(ਦਸਤਖਤ / ਖੱਬੇ ਅੰਗੂਠੇ ਦਾ ਪ੍ਰਭਾਵ) ਸਥਾਨ:

ਭਾਗੀਦਾਰ ਦਾ ਨਾਮ: ____________________________________

ਪੁੱਤਰ / ਨੂੰਹ / ਪਤੀ / ਪਤਨੀ: __________________________________

ਪੂਰਾ ਡਾਕ ਪਤਾ: _____________________________________

ਇਹ ਪ੍ਰਮਾਣਿਤ ਕਰਨ ਲਈ ਹੈ ਕਿ ਉਪਰੋਕਤ ਸਹਿਮਤੀ ਮੇਰੀ ਮੌਜੂਦਗੀ ਵਿਚ ਪ੍ਰਾਪਤ ਕੀਤੀ ਗਈ ਹੈ.

. ------------------------------ ਤਾਰੀਖ਼:

ਪ੍ਰਮੁੱਖ ਜਾਂਚਕਰਤਾ ਦੇ ਦਸਤਖਤ

1) ਗਵਾਹ – 1 2) ਗਵਾਹ – 2

------------------------------- ----------------------

ਦਸਤਖਤ ਦਸਤਖਤ

ਨਾਮ: ਨਾਮ:

ਪਤਾ: ਪਤਾ:

**INVESTIGATOR CERTIFICATE**

I certify that all the elements including the nature, purpose and possible risks of the above study as described in this consent document have been fully explained to the subject. In my judgment, the participant possesses the legal capacity to give informed consent to participate in this research and is voluntarily and knowingly giving informed consent to participate.

Signature of the Investigator: ______________

Name of the Investigator: ___________________

Dated: __________

**PATIENT PROFORMA**

Name: _________________________________ Age: _______ Sex: ________

Cr. No.: __________________ Special Clinic No.: ___________

Address: __________________________________________________

Phone No.: ___________________ Date of presentation:

**History**

1. Chief symptoms:-

2. Duration of symptoms:-

3. Treatment History:-

4. Associated Illness (Trauma, Myopia, Hypertension, etc.)

**Diagnosis**: OD _______________________________________________

OS ________________________________________________

**Plan**: ____________________________________________

# OCULAR EXAMINATION DATE

| Visual acuity | RE LE |
| --- | --- |
| Slit-lamp biomicroscopy  Mackie stage of neurotrophic keratopathy | RE LE |
| Fundus examination | RE LE |
| Facial asymmetry | RE LE |

# INVESTIGATIONS DATE

| Anesthsiometery | RE 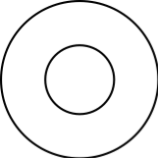 LE 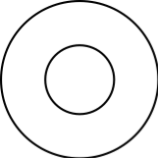 |
| --- | --- |
| TBUT | RE LE |
| Schirmer’s 1 | RE LE |

CONFOCAL MICROSCOPY

| Sub-basal nerve plexus | RE | LE |
| --- | --- | --- |
| No of nerve fibers/field | RE | LE |
| Nerve fiber density | RE | LE |

**Annexure I: MACKIE cLASSIFICATIOn**

| **STAGE** | - **CLINICAL FEATURES** | **TREATMENT** |
| --- | --- | --- |
| **1** | - Inferior palpebral conjunctiva staining with Rose Bengal (earliest sign) - Decreased TBUT - Increased mucous viscosity - Punctate epithelial fluorescein staining - Scattered small facets of dried epithelium(Gaule spots) | Lubricating Eye Drops |
| **2** | - Epithelial defect surrounded by a rim of loose epithelium - Stromal swelling with Descemet membrane folds - Edges of defect become smooth & rolled with time - Rare anterior chamber inflammatory action. - Recurrent PED | Artificial tears, lubricant ointments  Therapeutic soft contact lenses or patching  Topical autologous serum application,  Amniotic membrane grafting,  Tarsorrhaphy or botulinum induced ptosis  Topical Nerve Growth Factor application |
| **3** | - Corneal ulcer - Stromal Melting /Lysis - Corneal Perforation | Therapy for Stage 1 & 2 with  N-acetylcysteine,oral tetracycline,and medroxyprogesterone |

**REFERENCES :**

1.Benítez del Castillo JM, Wasfy MA, Fernandez C, Garcia-Sanchez J. An in vivo confocal masked study on corneal epithelium and subbasal nerves in patients with dry eye. Invest Ophthalmol Vis Sci 2004; 45: 3030–3035.

2. Garcia-Hirschfeld J, Lopez-Briones L, Belmonte C. Neurotrophic influences on corneal epithelial cells. Exp Eye Res 1994; 59: 597–605.

3. Baker KS, Anderson SC, Romanowski EG, Thoft RA, SundarRaj N. Trigeminal ganglion neurons affect corneal epithelial phenotype: influence on type VII collagen expression in vitro. Invest Ophthalmol Vis Sci 1993; 34: 137–144.

4. Heigle TJ, Pflugfelder SC. Aqueous tear production in patients with neurotrophic keratitis. Cornea. 1996;15:135–138.

5. Ueno H, Ferrari G, Hattori T, et al. Dependence of corneal stem/ progenitor cells on ocular surface innervation. Invest Ophthalmol Vis Sci. 2012;53:867–872.

6. Dua HS, Said DG, Messmer EM, *et al*. Neurotrophic keratopathy. *Prog Retin Eye Res* 2018

7. Rosenberg ML. Congenital trigeminal anesthesia: A review and classification. *Brain* 1984;107:1073–1082.

8. Mackie |A. Neuroparalytic keratitis. In: fraunfelder F, Roy FH, Meyer SM, eds.Current Ocular Therapy. Philadelphia, PA: WB Saunders; 1995:452-4

9. Ferrari G, Hajrasouliha AR, Sadrai Z, Ueno H, Chauhan SK, Dana R. Nerves and neovessels inhibit each other in the cornea. Investigative ophthalmology & visual science 2013;54:813-20.

10.Ueno H, Ferrari G, Hattori T, et al. Dependence of corneal stem/progenitor cells on ocular surface innervation. Investigative ophthalmology & visual science 2012;53:867-72

11. Pushker N, Dada T, Vajpayee RB, et al. Neurotrophic keratopathy. CLAO J. 2001;27:100–107.

12. Bonini S, Rama P, Olzi D, et al. Neurotrophic keratitis. Eye (Lond). 2003;17:989–995.

13. Terzis JK, Dryer MM, Bodner BI. Corneal neurotization: a novel solution to neurotrophic keratopathy. PlastReconstr Surg. 2009;123:112–120.

14. Fung SSM, Catapano J, Elbaz U, et al. In vivo confocal microscopy reveals corneal reinnervation after treatment of neurotrophic keratopathy with corneal neurotization. Cornea. 2018;37:109–112.

15. Elbaz U, Bains R, Zuker RM, et al. Restoration of corneal sensation with nerve transfers and nerve grafts: a new approach to a difficult problem. JAMA Ophthalmol. 2014;132:1289–1295.

16. Kauffmann T, Bodanowitz S, Hesse L, Kroll P. Corneal reinnervation after photorefractive keratectomy and laser in situ keratomileusis: An in vivo study with a confocal video- microscope. *Ger J Ophthalmol.* 1996;5:508–512.

17.Fuchsluger TA, Steuhl KP, Meller D. Neurotrophic kera- topathy: A post-LASIK case report [in German]. *KlinMonatsblAugenheilkd.* 2005;222:901–904.

18.Gallar J, Acosta MC, Moilanen JAO, Holopainen JM, Bel- monte C, Tervo TMT. Recovery of corneal sensitivity to mechanical and chemical stimulation after laser in situ kerato mileusis. *J Refract Surg.* 2004;20:229–235.

19. Adam M. Sonabend et al. [Schmidek and Sweet Operative Neurosurgical Techniques (Sixth Edition)](https://www.sciencedirect.com/book/9781416068396), 2012.

20. Samii M. Autologe Nerven-Transplantation im Trigeminusbereich. Med Mitt1972;46:189–94.

21. Samii M. Reconstruction of the trigeminal nerve. In: Samii M, Jannetta PJ, The cranialnerves. Berlin Heidelberg New York: Springer-Verlag, 1981:352–8.

22. Allevi F, Fogagnolo P, Rossetti L. Eyelid reanimation, neurotisation, and transplantation of the cornea in a patient with facial palsy. BMJ Case Rep 2014:pii: bcr2014205372.

23. Jacinto F, Espana E, Padilla M, et al. Ipsilateral supraorbital nerve transfer in a case of recalcitrant neurotrophic keratopathy with an intact ipsilateral frontal nerve: a novel surgical technique. Am J Ophthalmol Case Rep 2016;4:14–17.

24. 2018. Available from: http://www. sicsso. org/ video/ watch. php? vid= a0f635826

25. Leyngold I, Weller C, Leyngold M, et al. Endoscopic corneal neurotization: technique and initial experience. Ophthalmic Plast Reconstr Surg 2018;34:82–5.

26. Ting DSJ, Figueiredo GS, Henein C, et al. Corneal neurotization for neurotrophic keratopathy. Cornea 2018;37:641

27. Bains RD, Elbaz U, Zuker RM, et al. Corneal neurotization from the supratrochlear nerve with sural nerve grafts: a minimally invasive approach. Plast Reconstr Surg 2015;135:397e–400.

28. Sepehripour S, Lloyd MS, Nishikawa H, et al. Surrogate outcome measures for corneal neurotization in infants and children. J Craniofac Surg 2017;28:1167–70.

29. Weis E, Rubinov A, Al-Ghoul AR, et al. Sural nerve graft for neurotrophic keratitis: early results. Can J Ophthalmol 2018;53:24–9.

30. Joseph catapano et al. Treatment of Neurotrophic Keratopathy with Minimally Invasive Corneal Neurotisation: Long-Term Clinical Outcomes and Evidence of Corneal Reinnervation. Br J Ophthalmol 2019 Dec;103:1724-1731.doi: 10.1136/bjophthalmol-2018-313042.

31. Asbell PA, Lemp MA. Dry eye disease: The clinician’s guide to diagnosis and treatment, 2009:39

32.P.J. Murphy et al. Reliability of the Non-Contact Corneal Aesthesiometer. Ophthal. Physiol. Opt. 1998 18: No 6.

33.Pauline Cho et al.Tear break-up time: clinical procedures and their effects. Ophthal. Physiol. Opt. 1998 18: No.

34.Vanathi M, Tandon R, Sharma N, Titiyal JS, Pandey RM, Vajpayee RB. In-vivo slit scanning confocal microscopy of normal corneas in Indian eyes. Indian J Ophthalmol 2003; 51: 225-230.

35.Matthias Bohnke et al.Confocal microscopy of the cornea. Progress in the retina and eye research 1999;18:570-71

36. Oliveira-Soto LM, Efron N. Morphology of cornea nerves using confocal microscopy. Cornea 2001; 20: 374-384.

37. C. E. Wisely et al. Clinical and Morphologic Outcomes of Minimally Invasive Direct Corneal Neurotization. Ophthalmic Plastic and Reconstructive Surgery. February 2020.
