# Supplementary material for: Clinical outcomes of corneal neurotization using sural nerve graft in neurotrophic keratopathy
Source: PLoS One. 2023 Nov 28;18(11):e0294756. doi: 10.1371/journal.pone.0294756 (PMC10684005; doi:10.1371/journal.pone.0294756)
Supplement: Attachment — Submitted filename: Response to Reviewers .docx [file pone.0294756.s005.docx]

**Dear Editor-in-chief,**

PLOS ONE

We appreciate the thorough review of our manuscript PONE-D-23-06245R1 titled **"Clinical Outcomes of Corneal Neurotization Using Sural Nerve Graft in Neurotrophic Keratopathy** " and the valuable feedback provided by the editor and reviewers.

We believe that these revisions enhance the overall quality and accuracy of our manuscript

**JOURNAL REQUIREMENTS:**
Please review your reference list to ensure that it is complete and correct. If you have cited papers that have been retracted, please include the rationale for doing so in the manuscript text, or remove these references and replace them with relevant current references. Any changes to the reference list should be mentioned in the rebuttal letter that accompanies your revised manuscript. If you need to cite a retracted article, indicate the article’s retracted status in the References list and also include a citation and full reference for the retraction notice.

**Response-** We have meticulously reviewed our reference list to ensure its completeness and accuracy. **Reference 4 and 6,** which were not identified in the PubMed search **have been replaced with the relevant and appropriate citations.**

**REVIEW COMMENTS TO THE AUTHOR**

**Reviewer #1**

We sincerely appreciate your thorough review of our manuscript. Your feedback has been invaluable in improving the quality of our work. We have carefully considered your comments and made the necessary revisions accordingly.

**Comment-:** Table 3 better report median (IQR or min max) since nonparametric method was used. Flowchart, there are still two arms. This is one-arm study. Two-arm chart usually refers to two treatment groups.
The one year follow up box should be put underneath the 6 month box in one line.

**Response-** We are grateful for your thoughtful input, which has undoubtedly improved the quality of our manuscript. We have **updated the table 3 to report the median along with the interquartile range (IQR) to** provide a more accurate representation of the data.

We acknowledge the confusion created by the two-arm chart representation. Therefore, we have corrected the error and updated the flowchart to clearly reflect the one-arm study design, eliminating any ambiguity in the visual presentation

Thank you for pointing out the arrangement of the timeline boxes. **We have rearranged the flow chart follow up depiction**, one-year follow-up box beneath the 6-month box to ensure that the flowchart now accurately represents the temporal sequence of the study.

**Reviewer #3:**

We truly appreciate your diligence in reviewing our manuscript and your insightful comment on the statistical analysis section. Your expertise has been invaluable in identifying the appropriate statistical method for our study. We believe that these changes have strengthened the overall presentation and interpretation of our study findings.

**Comment-** The main requirement is regarding the statistical subsection. Fisher`s exact test is recommended for categorical data, and NK grade severity is ordinal, so the more suitable statistical method in the before-after empirical situation is Wilcoxon signed rank test. So, there is need to reword the part of statistical analysis that talks about the usage of Wilcoxon signed rank test like this: “Wilcoxon signed rank test was used for …” and to change the results regarding NK grade severity and follow-up.

**Response-** In response to your suggestion, we have carefully considered the statistical analysis and **used the Wilcoxon signed rank test for before and after corneal neurotization to asses NK grade severity.**

Consequently, we have reworded the statistical analysis section to accurately reflect the utilization of the Wilcoxon signed rank test for assessing the changes in NK grade severity.

Furthermore, we have recalculated and updated the results related to NK grade severity and follow-up in table 2 (Yellow highlighted). This modification ensures the statistical integrity and consistency of our study.

We are grateful for your meticulous attention to detail and your guidance in enhancing the accuracy of our research. Your insights have contributed significantly to the overall quality of the manuscript.

Thank you for your time and commitment to improving our work.

**Sincerely**

Prof. Arun K. Jain

Professor and Head of Cornea, Cataract and Refractive Surgery

Advanced Eye Centre, Post Graduate Institute of Medical Education and

Research, Chandigarh-160012, India.
